# Association of *NOTCH3* Variant Position With Stroke Onset and Other Clinical Features Among Patients With CADASIL

**DOI:** 10.1212/WNL.0000000000200744

**Published:** 2022-08-02

**Authors:** Bernard P.H. Cho, Amy A. Jolly, Stefania Nannoni, Daniel Tozer, Steven Bell, Hugh S. Markus

**Affiliations:** From the Department of Clinical Neurosciences, University of Cambridge, United Kingdom.

## Abstract

**Background and Objectives:**

Cerebral autosomal dominant arteriopathy with subcortical infarcts and leukoencephalopathy (CADASIL) is caused by a cysteine-altering variant in 1 of the 34 epidermal growth factor-like repeat (EGFR) domains of the NOTCH3 protein. CADASIL has a variable phenotypic presentation, and *NOTCH3* variants in EGFRs 1–6 have been found correlated with greater disease severity. We examined clinical and radiologic features and performed bioinformatic annotation of variants in a large CADASIL cohort to further understand these associations.

**Methods:**

We examined the association of *NOTCH3* variant position on stroke onset and other clinical features among patients with CADASIL from the United Kingdom. We also explored how in silico predicted protein aggregation differed by variant position and the extent to which this affected stroke risk.

**Results:**

We identified 76 different cysteine-altering *NOTCH3* variants in our cohort of 485 patients (mean age: 50.1 years; % male: 57.5). After controlling for cardiovascular risk factors, variants in EGFRs 1–6 were associated with earlier onset of stroke (hazard ratio [HR]: 2.05, 95% CI: 1.43–2.94) and encephalopathy (HR: 2.70, 95% CI: 1.15–6.37), than variants in EGFRs 7–34. Although the risk of stroke was higher in the patients with predicted protein aggregation (HR: 1.50, 95% CI: 1.05–2.14), this association was no longer significant after controlling for variant site. Further analysis suggested that lower stroke risk was observed for variants in EGFRs 10–17 compared with variants in the other EGFR domains.

**Discussion:**

*NOTCH3* variant position is a predictor of stroke and encephalopathy in CADASIL independent of cardiovascular risk factors. Lower stroke risk was found for variants in EGFRs 10–17. Molecular factors that influence CADASIL disease severity remain to be determined.

Cerebral autosomal dominant arteriopathy with subcortical infarcts and leukoencephalopathy (CADASIL) is the most common genetic form of stroke, resulting in early onset lacunar stroke and dementia.^1,2^ It is caused by characteristic cysteine-altering variants in the epidermal growth factor-like repeat (EGFR) domains of the *NOTCH3* gene.^3^ The clinical phenotype is highly heterogeneous with stroke onset ranging from the fourth to eighth decade.^4^

The reasons for this variation remain uncertain. Conventional cardiovascular risk factors, particularly smoking and hypertension, are associated with an earlier age at stroke onset, but account for only a small part of the phenotypic variation.^5,6^ Previous studies reported no association between variant site and phenotype. However, recently, it was suggested that variants located in EGFR domains 1–6 are associated with more severe disease than variants in domains 7–34, including earlier age at stroke onset, shorter survival, and increased white matter hyperintensity (WMH) volume.^7^ The initial study reported associations with age at onset of stroke in 251 Dutch patients with CADASIL, but these were yet to be confirmed in other ethnic groups of patients with CADASIL. It has also been separately suggested that variants in the ligand-binding site (EGFR domains 10 and 11) may be associated with more severe disease.^8^

In a large cohort of 485 individuals with CADASIL presenting to a national CADASIL clinic in the United Kingdom, we attempted to replicate, and further delineate, associations between variant site and both clinical and radiologic phenotypes. We assessed whether variants in EGFR domains 1–6, and in the ligand-binding site, were associated with more severe disease. We also investigated the best cutoff to separate patients into high-risk and low-risk groups of stroke based on variant site. We further examined the effects of the CADASIL variants on aggregation propensity to investigate the molecular mechanisms underlying variability in disease severity among patients.

## Methods

### Data Collection

Longitudinal data were collected from patients with CADASIL over a 30-year period, as part of a UK CADASIL National Referral Service from 1990 to 2015 and through enrolment to the UK Familial Cerebral Small Vessel Disease Study from 2016 to 2021. Data from both sources were collected through a uniform approach on a standard proforma.

485 patients were included in this study. They were evaluated and examined by consultant neurologists as part of their clinical appointment. The collected data were reviewed by a neurologist, and major disease events were confirmed by referring to original hospital records.

All the patients had confirmed genetic diagnosis through direct sequencing of the *NOTCH3* gene to identify the pathogenic variants for CADASIL, namely missense variants, leading to the gain or loss of a cysteine residue in 1 of the 34 EGFR domains of the NOTCH3 protein. Patients who were diagnosed with CADASIL by presymptomatic genetic testing were also included (n = 22). Two patients with confirmed homozygous or compound heterozygous variants on the *NOTCH3* gene were excluded because they may have different clinical features that mask the effects of variant position on disease severity.^9^

### Data Fields

The following fields were collected for all patients: demographics (year of birth and sex), *NOTCH3* variant information, first presenting feature and age at which it was experienced, history of and age at onset of various diseases and symptoms (such as stroke and encephalopathy), and history of cardiovascular risk factors (namely hypertension and smoking). Brain metrics, including total brain volume, WMH volume, lacune count, and microbleed count were available if the patient had a clinical MRI. Brain and WMH volumes were recorded in cm^3^.

Stroke was diagnosed based on clinical and neuroradiologic assessment and defined as a focal neurologic event lasting more than 24 hours accompanied by brain imaging evidence. Migraine was defined by the 2004 International Classification of Headache Disorders.^10^ Epileptic seizures were only included if they occurred spontaneously and not as part of an encephalopathic episode. History of any psychiatric illness, hypertension, hypercholesterolemia, or diabetes was recorded. CADASIL encephalopathy was defined as in a previous article as an acute reversible encephalopathy with evidence of reduced consciousness in the absence of any other organic cause, where symptoms lasted for longer than 24 hours and were sufficient to warrant hospital admission.^11^ History of depression, defined as depression requiring treatment with drug or psychological therapy, and symptomatic cognitive impairment was determined from self-reported records taken during clinical assessment. Hypertension was defined as a systolic blood pressure above 140 mm Hg and/or a diastolic pressure above 90 mm Hg or use of antihypertensive medication. Hypercholesterolemia was defined as a serum cholesterol greater than 5.2 mmol/L or on lipid-lowering drugs.

### Standard Protocol Approvals, Registrations, and Patient Consents

All participants in this study gave written informed consent. The UK Cerebral Small Vessel Disease Study was approved by the East of England–Cambridge Central Research Ethics Committee (Ref: 16/EE/0118).

### Brain Imaging Analysis

Brain MRI scans acquired during routine clinical management were available for some patients. Some of the scans were excluded from further analysis for each of the MRI parameters studied (WMH volume, lacune, and microbleed quantification) because of quality issues and/or unavailable MRI sequence. MRI was performed at the patient's local hospital at field strengths of between 0.5 and 3 T. T1-weighted and fluid-attenuated inversion recovery (FLAIR) T2-weighted sequences were used to calculate brain and WMH volumes, respectively.

Brain volumes were estimated using SIENAX^12^ from the FMRIB's software library 5.0.9.^13^ Brain tissue was extracted using an automated Brain Extraction Tool^14^ and linearly registered using affine transformation,^15,16^ which helps to normalize volumes for skull size. Tissue-type segmentation of images was performed through a hidden Markov random field model and the expectation-maximization algorithm.^17^ Normalized measures of gray matter, white matter, and total brain volumes were calculated by multiplying the measured volume by its estimated scaling factor.

Quantification of WMH on FLAIR images was performed using a semiautomated contouring method in the JIM software package version 8.0 (Xinapse Systems, Colchester, United Kingdom). WMH volumes were marked and measured on T2 FLAIR sequences in the axial plane by a trained researcher. Final volumes were multiplied by the scaling factor derived from the brain volume to account for skull size. The WMH lesion volumes were marked on 10 randomly selected MRI scans by 2 independent raters. The intraclass correlation (ICC) between the 2 raters was 0.96.

Lacunes and microbleeds were quantified by a neurologist. Lacunes were manually counted on FLAIR images and distinguished from perivascular spaces based on their location, size, and shape on T1-weighted and T2-weighted scans. Lacunes were counted if they were in the subcortex and larger than 3 mm in diameter. Microbleeds were counted using the Brain Observer Microbleed Scale, defined as round well-defined hypointense foci on susceptibility weighted imaging or gradient echo sequences with a diameter of 2–10 mm.^18^ The inter-rater agreement for lacune and microbleed counts were good, ICC for count data = 0.91 and 0.99, respectively.

### Molecular Annotation of *NOTCH3* Variants

Several bioinformatic tools were used to predict the molecular effects of *NOTCH3* variants at different positions. Variants were annotated using Ensembl Variant Effect Predictor.^19^ They were mapped onto protein 3-dimensional structures in the Protein Data Bank on VarSite,^20^ whose information was analyzed to predict the molecular effects of the variants. Protein aggregation scores were calculated based on a small part of the NOTCH3 protein structure using the Aggrescan3D web server version 2.0,^21^ with the distance of aggregation analysis specified at 5 Å. The intrinsic aggregation propensity of each amino acid was computed and used to generate the average aggregation score of the protein structure. The difference in residue aggregation scores between the mutated NOTCH3 protein and the wild-type protein was calculated for each *NOTCH3* variant. Proaggregation variants were defined as variants causing an increased residue score (>0).

### Statistical Analyses

Baseline characteristics and cardiovascular risk factors were compared between patients with variants in EGFRs 1–6 and 7–34 by 2-sample *t* test. Any variables that did not follow a normal distribution were transformed: Brain and WMH volumes were natural-log transformed; lacune and microbleed counts were square rooted. The association of EGFR group with risk of various clinical features was analyzed by logistic regression with adjustment for age, sex, hypertension, and smoking, and the effects of variant position on MRI measures were analyzed by linear regression with adjustment for age, sex, and MRI field strength. Kaplan-Meier analyses and Cox proportional hazards regression (using age as the underlying time scale) with adjustment for sex and cardiovascular risk factors were performed to compare the time of onset of disease in different EGFR groups. These analyses incorporated all patients and censored those who had not experienced an event at the time of their most recent clinical assessment.

Our initial a priori analysis was based on previous literature suggesting EGFRs 1–6 variants had a more severe phenotype than EGFRs 7–34 variants.^7^ After this comparison, further exploratory analyses were performed with *NOTCH3* variants that were further divided based on groupings other than EGFRs 1–6 and 7–34 to investigate whether any other EGFR cutoff better separated patients into high-risk and low-risk groups for stroke. Variants in EGFRs 1–6 were also subdivided into 3 smaller groups (EGFRs 1–2, 3–4, and 5–6) to determine whether associations were stronger within any subdivision of this EGFR category. The hazard ratios (HRs) of stroke for these groups were calculated using the Cox proportional hazards model, where assumptions were tested based on Schoenfeld residuals, and no violations were observed. We used floating variances to estimate 95% CIs for each group (including the reference group) that reflect the amount of information underlying each category.^22^

The association of gain-of-cysteine and proaggregation variants on stroke outcomes was investigated. Both types of variants were correlated with EGFR position (1–6 vs 7–34) using logistic regression (adjusted for age and sex) and with the age at onset of the first stroke using Cox regression (adjusted for sex and EGFRs 1–6 variant). To assess whether gain-of-cysteine and proaggregation variants mediated the effect of EGFR position on stroke, mediation analyses were performed using the “mediation” package in R.^23^ Standard errors of the indirect effect (i.e., the association of variant on risk of stroke acting through the molecular mediator considered) were obtained using 10,000 bootstrap samples.

All statistical analyses were performed using R V.4.0.3 with 2-sided *p* values, and *p* < 0.05 considered statistically significant.

### Data Availability

The patient data used for this study are available from the corresponding author on reasonable request.

## Results

### Distribution of Risk Factors and *NOTCH3* Variants

A total of 485 patients from 345 different families with a genetically confirmed CADASIL diagnosis were included in this study. All patients carried heterozygous *NOTCH3* variants. The demographic and risk factor profile of patients are summarized in eTable 1 (links.lww.com/WNL/C74).

**Figure 1:**
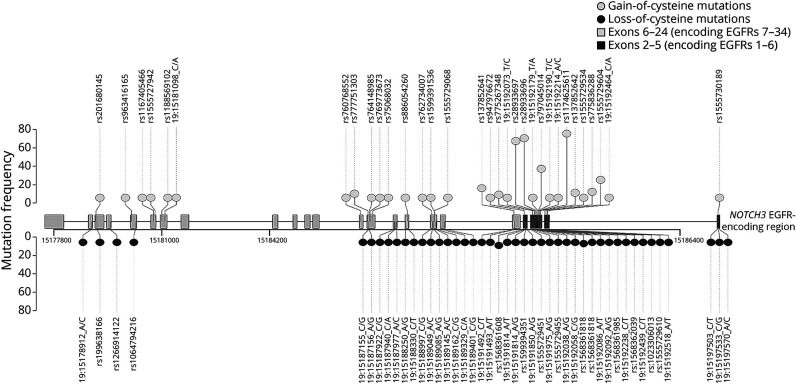
Lolliplot Showing the Distribution of Distinct Variants Present in the Patient Cohort Across the Reverse Strand of the *NOTCH3* Gene Lighter circles represent gain-of-cysteine variants; darker circles represent loss-of-cysteine variants. Lighter rectangular boxes represent the exons that encode EGFRs 7–34. Darker rectangular boxes represent the exons that encode EGFRs 1–6. EGFR = epidermal growth factor-like repeat.

There were 76 different *NOTCH3* variants spread across 16 different exons (between exons 2 and 24 of the *NOTCH3* gene), with most in exons 3 and 4 (n = 358, 76.8%) (Figure 1 and eTable 2, links.lww.com/WNL/C75). Most of the variants were in the proximal region of *NOTCH3* encoding EGFRs 1–6 (n = 391, 80.6%, see eFigure 1, links.lww.com/WNL/C71). Variants in EGFRs 3 and 4 comprised 28.7% (n = 139) and 32.4% (n = 157) of the cohort, respectively. Thirty-two of 76 (42.1%) variants led to the gain of cysteine residue in an EGFR domain, whereas 44 (57.9%) led to the loss of a cysteine (Figure 1 and eTable 2).

### Variants in *NOTCH3* EGFR Domains 1–6 Are Associated With Earlier Onset of Stroke and Encephalopathy

Patients with an EGFRs 1–6 variant were on average 5.1 years younger than patients with an EGFRs 7–34 variant (49.1 years vs 54.2 years, *p* = 0.0002) (eTable 1, links.lww.com/WNL/C74). After adjusting for age and sex, variants in EGFRs 1–6 were associated with a more than twofold increase in the odds of stroke (odds ratio [OR]: 2.09, 95% CI: 1.27–3.49, *p* = 0.003) (Table 1).

**Table 1:**
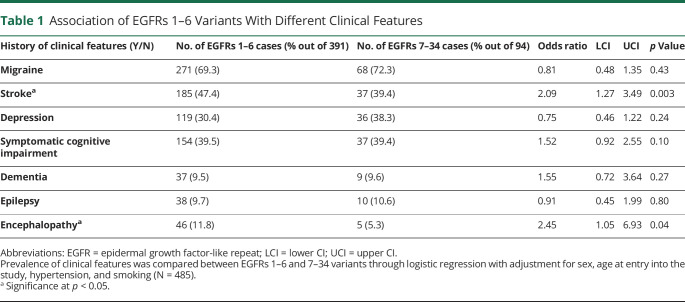
Association of EGFRs 1–6 Variants With Different Clinical Features

**Figure 2:**
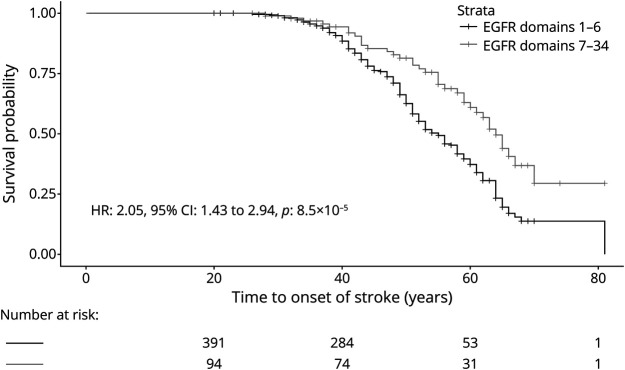
Kaplan-Meier Plot Showing the Difference in Age at Onset of the First Stroke for Patients With EGFR Domain 1–6 Variants and Those With EGFR Domain 7–34 Variants HR was calculated through Cox regression with adjustment for sex and cardiovascular risk factors. EGFR = epidermal growth factor-like repeat; HR = hazard ratio.

Median age at onset of the first stroke was 55 (interquartile range [IQR]: 13) years for EGFRs 1–6 patients and 64 (IQR: 18) for EGFRs 7–34 patients. Using Cox regression after adjustment for sex and cardiovascular risk factors, variant location remained a strong predictor of age at stroke onset (HR for EGFRs 1–6 variant vs EGFRs 7–34 variant: 2.05, 95% CI: 1.43–2.94, *p* = 8.5 × 10^−5^) (Figure 2). Family of origin did not alter the effect of *NOTCH3* variant position on age at stroke onset (i.e., with the inclusion of only 1 patient per family in our analysis, similar results were found).

**Figure 3:**
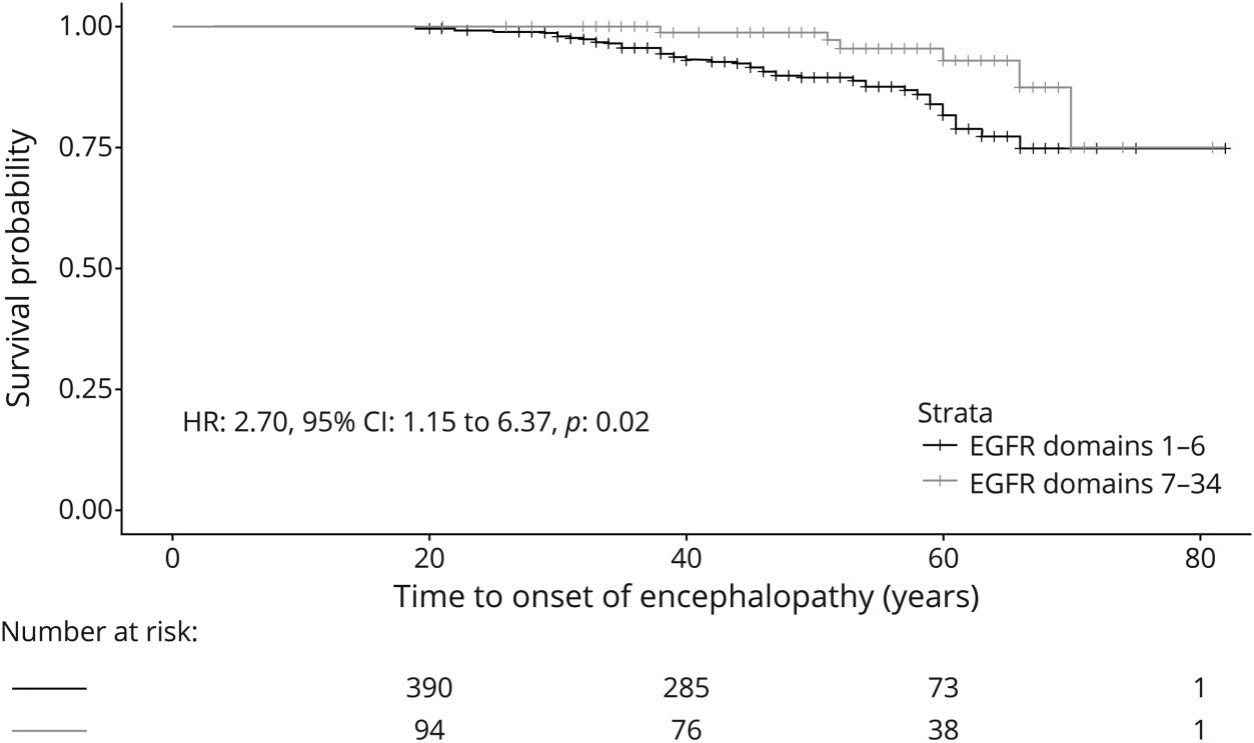
Kaplan-Meier Plot Showing the Difference in Age at Onset of the First Encephalopathy for Patients With EGFR Domain 1–6 Variants and Those With EGFR Domain 7–34 Variants HR was calculated through Cox regression with adjustment for sex and cardiovascular risk factors. EGFR = epidermal growth factor-like repeat; HR = hazard ratio.

Encephalopathy was more prevalent in the patients with an EGFRs 1–6 variant than those with an EGFRs 7–34 variant (OR: 2.45, 95% CI: 1.05–6.93, *p* = 0.04) (Table 1). In Cox models, after adjustment for sex and cardiovascular risk factors, EGFRs 1–6 variants were associated with age at first encephalopathy (HR: 2.70, 95% CI: 1.15–6.37, *p* = 0.02) (Figure 3).

### Effects of Variant Position on Brain MRI Measures

MRI scans from 206 patients with an EGFR 1–6 variant and 55 with an EGFR 7–34 variant were visually rated and compared. For each MRI marker, the numbers of analyzable scans were brain volume (205), WMH (206), lacunes (199), and microbleeds (129). At the time of the MRI scans, EGFR 1–6 patients were on average 6.6 years younger than EGFR 7–34 patients (48.4 vs 55.0 years, *p* = 0.00008). In unadjusted analyses, there was a borderline association between *NOTCH3* variant position and WMH (*R* = −0.12, *p* = 0.05) (eFigure 2, links.lww.com/WNL/C72); however, no differences were found when comparing the MRI markers between the 2 groups of patients (Table 2). The results were similar before and after adjustment for MRI field strength, which only correlated with microbleeds (β: 0.18, 95% CI: 0.03–0.33, *p* = 0.02).

**Table 2:**
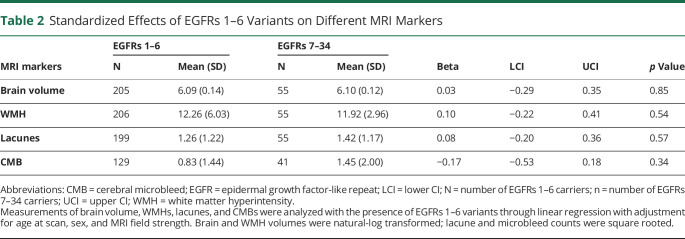
Standardized Effects of EGFRs 1–6 Variants on Different MRI Markers

### Do Other EGFR Cutoffs Better Identify Cases With an Higher Risk of Stroke?

**Figure 4:**
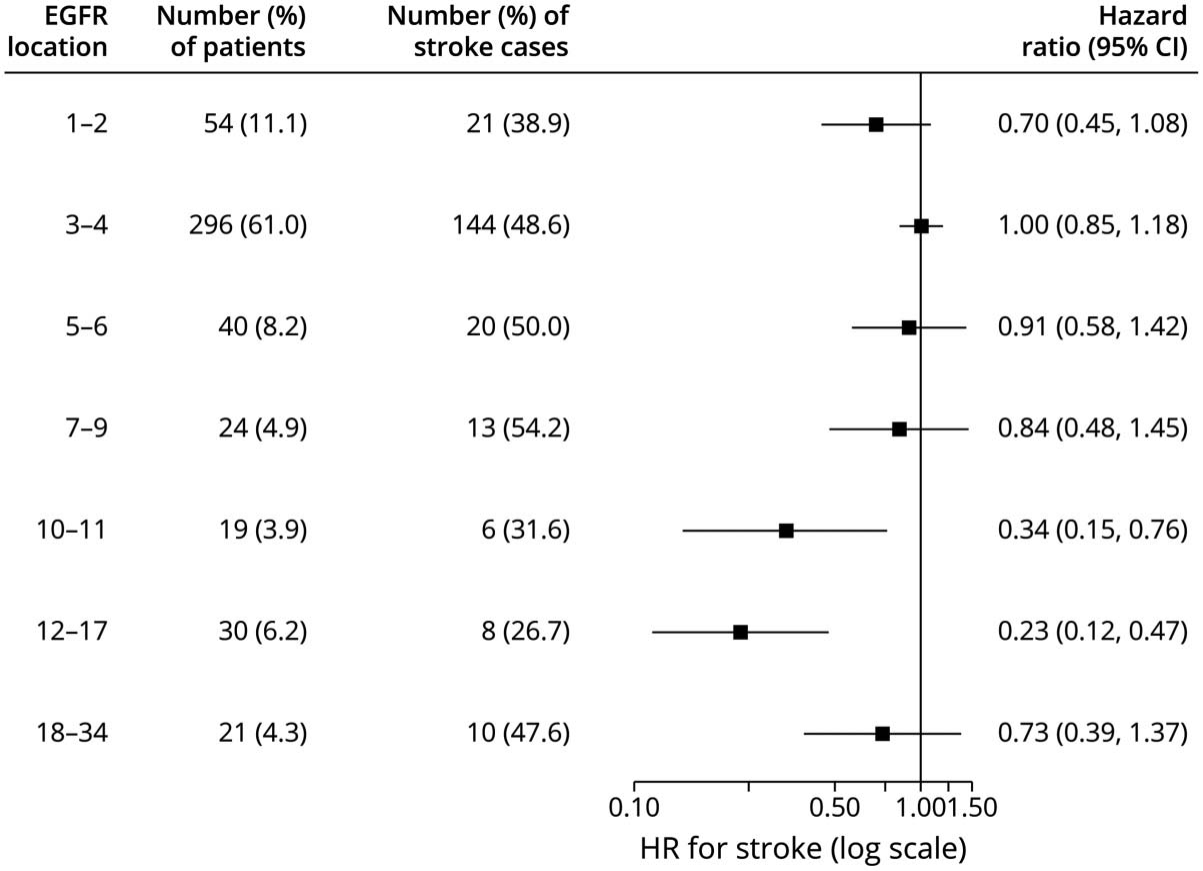
Forest Plot Showing the Risk of Stroke Associated With Variants in Different EGFR Location Compared With EGFRs 3–4 Prevalence of stroke cases was analyzed with the presence of variants in different EGFR location through Cox regression, with the EGFRs 3–4 variant group being used as the reference and with adjustment for sex and cardiovascular risk factors (N = 485). EGFR = epidermal growth factor-like repeat; HR = hazard ratio.

The effects of different EGFRs cutoffs other than 1–6 and 7–34 on the presence of stroke were examined. Figure 4 shows the association of variants in different EGFR regions with risk of stroke. This illustrates that there was no clear difference in strength of association with stroke for EGFRs 1–9 and 18–34 but that EGFRs 10–17 seemed to be associated with a lower stroke risk compared with domains 3–4 (Figure 4). When using EGFRs 3–4 as the reference, the risk of stroke associated with EGFRs 12–17 was almost 80% lower (HR: 0.23, 95% CI: 0.12–0.47, *p* = 7.7 × 10^−5^).

Variants in the ligand-binding site (EGFRs 10–11) were also associated with a lower risk of stroke compared with variants in EGFRs 3–4 (HR: 0.34, 95% CI: 0.15–0.76, *p* = 0.01). MRI markers did not differ between patients with an EGFRs 10–11 variant and those with a variant outside EGFRs 10–11 (eTable 3, links.lww.com/WNL/C76).

### Molecular Factors That May Influence CADASIL Disease Severity

**Figure 5:**
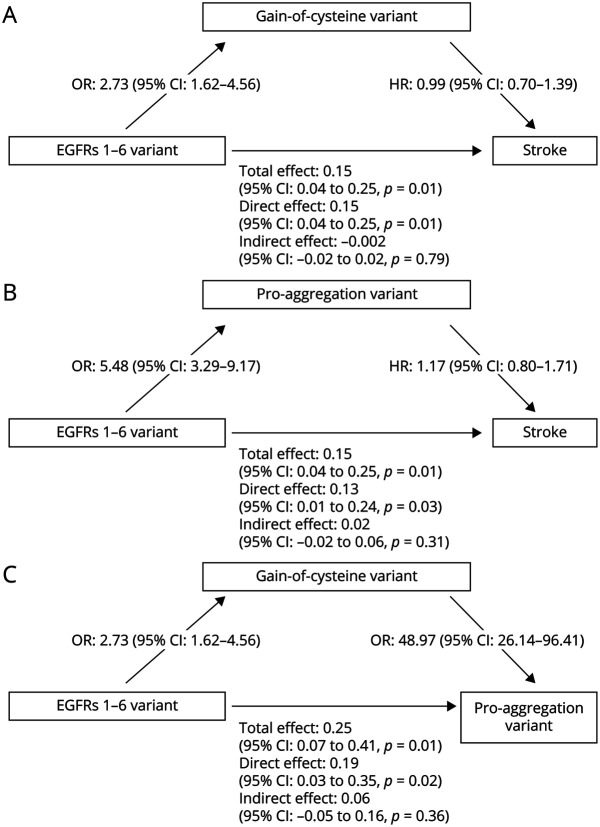
Mediation Analyses (A) Effect of the presence of gain-of-cysteine variant on the relationship between EGFRs 1–6 variant and stroke. (B) Effect of the presence of proaggregation variant on the relationship between EGFRs 1–6 variant and stroke. (C) Effect of the presence of gain-of-cysteine variant on the relationship between EGFRs 1–6 variant and proaggregation variant. OR was calculated through logistic regression with adjustment for age and sex. HR was calculated through Cox regression with adjustment for sex and EGFRs 1–6 variants. Regression models were bootstrapped 10,000 times to examine whether a mediation effect was statistically significant. EGFR = epidermal growth factor-like repeat; HR = hazard ratio; OR = odds ratio.

Variants in EGFRs 1–6 were more likely to be gain-of-cysteine than loss-of-cysteine (OR: 2.73, 95% CI: 1.62–4.56, *p* = 0.0002). The mean age at onset of the first stroke for the gain-of-cysteine variant group did not differ from the others (restricted mean (median), 57.7 years (57 years) vs 58.8 years (59 years), *p* = 0.40). After adjustment for sex, no association was found between gain-of-cysteine variants and stroke onset (HR: 1.14, 95% CI: 0.82–1.60, *p* = 0.44). The HR remained nonsignificant after further adjusting for family of origin, cardiovascular risk factors, and EGFR group (HR: 1.07, 95% CI: 0.72–1.60, *p* = 0.74). Consistent with this, formal mediation analysis indicated that gain-of-cysteine variant did not mediate the effect of variant site on stroke risk (indirect effect: −0.002, 95% CI: −0.02 to 0.02, *p* = 0.79) (Figure 5A) or on age at stroke onset when only focusing on those who experienced a stroke (indirect effect: 0.19, 95% CI: −0.18 to 0.53, *p* = 0.51).

Earlier variant position was associated with a larger difference in residue aggregation scores between the mutated and wild-type protein. (eFigure 3, links.lww.com/WNL/C73). This is coherent with analyses which revealed EGFRs 1–6 variants were more likely to be proaggregation, that is, the variant was associated with a higher residue aggregation score than EGFRs 7–34 (OR: 6.13, 95% CI: 3.70–10.19, *p* = 3.2 × 10^−12^).

When focusing on aggregation status of variants, the mean age at onset of the first stroke for the proaggregation variant group (change in residue aggregation scores between the mutated and wild-type protein >0) was lower (restricted mean (median), 56.8 years (56 years) vs 62.7 years (60 years), *p* = 0.02). After adjustment for sex, proaggregation variants were associated with age at stroke onset (HR: 1.50, 95% CI: 1.05–2.14, *p* = 0.03). The HR remained significant after correction for family of origin and cardiovascular risk factors (HR: 1.63, 95% CI: 1.08–2.47, *p* = 0.003), but it became nonsignificant after controlling for EGFRs 1–6 (HR: 1.17, 95% CI: 0.80–1.71, *p* = 0.43). Furthermore, mediation analysis showed that proaggregation variant status did not mediate the effect of variant site on stroke risk (indirect effect: 0.02, 95% CI: −0.02 to 0.06, *p* = 0.31) (Figure 5B) or on age at stroke onset when only focusing on those who experienced a stroke (indirect effect: 0.39, 95% CI: −0.17 to 1.10, *p* = 0.17). Gain-of-cysteine variant did not mediate the effect of variant site on proaggregation variant status (indirect effect: 0.06, 95% CI: −0.05 to 0.16, *p* = 0.36) (Figure 5C). We also performed the analysis using the change in residue aggregation scores between the mutated and wild-type protein as a continuous variable instead of using the aggregation status of variants, and similarly, no evidence of mediation was found.

## Discussion

In this large study of 485 patients with CADASIL, we confirmed that variant site is a predictor of disease severity independent of cardiovascular risk factors, particularly regarding the age at onset of stroke.^7^ Our initial a priori analysis was based on previous literature suggesting EGFRs 1–6 variants had a more severe phenotype than EGFRs 7–34 variants,^7^ and we confirmed this observation with EGFRs 1–6 variants having earlier age at onset of both stroke and encephalopathy. Using our larger sample size, we then performed further exploratory analyses further dividing *NOTCH3* variants based on groupings other than EGFRs 1–6 and 7–34. Although most patients with CADASIL had a variant in EGFRs 1–6 (80.6% of our cohort and 71.7% of the Dutch cohort^7^), our results suggest that EGFRs 1–6 may not necessarily be the best cutoff to separate patients into high-risk and low-risk groups of stroke. EGFRs 1–9 and 18–34 seemed to be associated with a similar magnitude of stroke risk. Compared with EGFRs 3–4 variants, patients with variants in EGFRs 10–17 had a lower risk of stroke. Some previous reports have suggested that variants in the ligand-binding site (EGFRs 10–11) may be more severe^8^; however, we found no evidence to support this.

Although other studies have shown EGFRs 7–34 variants to be associated with very mild phenotype,^24-28^ EGFRs 18–34 variants seemed to be as severe as EGFRs 1–9 with respect to stroke in our study. However, there were fewer patients with variants in EGFRs 18–34 which reduces confidence in this comparison. Further studies are required to determine whether variants in EGFRs 18–34 are associated with a milder or similar phenotype, to variants in EGFRs 1–6.

It remains unclear why variants in certain EGFRs are associated with more severe disease. A recent study has demonstrated increased *NOTCH3* deposition in the skin biopsies of patients with variants in early EGFRs.^29^ One possible explanation is that they are associated with increased protein aggregation. We demonstrated that variants in earlier EGFR domains were associated with increased protein aggregation, as predicted by the in silico score; nonetheless, this did appear not mediate the effect of variant site on risk of stroke. However, notably, there are limitations to the aggregation score used in this study. For example, Aggrescan3D is yet to be validated, and we are uncertain about how sensitive and specific the score is in predicting aggregation of *NOTCH3* in different organs and tissues. In addition, the protein aggregation score was calculated based on only a partial NOTCH3 protein model that was available on VarSite. Further studies are needed to determine the effect of the variants on aggregation within specific tissues and how this affects disease severity before any firm conclusions can be drawn. We also investigated whether gain-of-cysteine and loss-of-cysteine affect disease severity. Although the gain-of-cysteine variants were strongly associated with variant position, they did not affect the age at stroke onset and therefore did not mediate the effect of variant site on stroke.

In contrast to strong associations between variant site and stroke risk, we found few associations between chronic markers of small vessel disease on MRI scans. There was a borderline association with WMH lesion volume, but no association with brain volume. It is possible that we were underpowered to detect associations with MRI markers because MRI was not available for all subjects. However, it is also possible that variant site is associated with a greater extent with certain disease manifestations, such as acute stroke, and less strongly associated with more chronic disease manifestations resulting in WMH and brain atrophy.^25^ Further studies in larger samples with MRI are needed to answer this question.

Much of the variability in disease severity is not explained by variant location. Cardiovascular risk factors, particularly hypertension and smoking,^30^ have been suggested to exacerbate the phenotype, but these also only explain a small part of disease variability. This raises the hypothesis that genetic modifiers outside the *NOTCH3* gene modify phenotypic expression. In fact, it has been estimated that in CADASIL variant carriers, the heritability of white matter lesion volume is as high as 60%–70% after accounting for *NOTCH3* variant site as well as age and sex.^31^ Consistent with this, a small genome-wide association study identified *APOE ε2* as a potential disease modifier.^32^ Further studies are warranted to identify genetic modifiers, outside the *NOTCH3* gene, which affects the penetrance of *NOTCH3* variants.

Our study has a number of strengths. First, it has a larger sample size than previous CADASIL cohorts, and we examined disease severity on MRI scans in more than 200 individuals. Second, all cases were reviewed in a single clinic by consultant neurologists on a prospective disease register using a standard proforma. Third, we correlated different variant sites with a variety of clinical features and showed associations with stroke were independent of conventional cardiovascular risk factors.

Our study also has limitations. MRI scans were clinically acquired on different scanners at different field strengths, and not all sequences were available for some patients. We controlled for field strength, and this did not alter the associations. However, because scans were performed on a wide range of scanners throughout the United Kingdom, there were not sufficient cases on the same scanner to allow us to determine when associations were similar when cases were confined to those on 1 scanner type. A second limitation is that because more patients with CADASIL have variants in proximal EGFR regions, we included a lower number of distal variants in the analysis.

In conclusion, our study confirms that *NOTCH3* variant position is a predictor of disease severity, particularly regarding the age at onset of stroke, and this is independent of conventional cardiovascular risk factors. We also reported an association with encephalopathy. Further research is needed to investigate the molecular mechanisms underlying the phenotypic differences associated with *NOTCH3* variant position.

## Glossary

CADASIL: cerebral autosomal dominant arteriopathy with subcortical infarcts and leukoencephalopathy
EGFR: epidermal growth factor-like repeat
FLAIR: fluid attenuated inversion recovery
HR: hazard ratio
ICC: intraclass correlation
IQR: interquartile range
OR: odds ratio
WMH: white matter hyperintensity
